# Theoretical Kinetic and Mechanistic Studies on the Reactions of CF_3_CBrCH_2_ (2-BTP) with OH and H Radicals

**DOI:** 10.3390/molecules22122140

**Published:** 2017-12-06

**Authors:** Huiting Bian, Lili Ye, Jinhua Sun

**Affiliations:** 1 State Key Laboratory of Fire Science, University of Science and Technology of China, Hefei 230026, China; bianht@mail.ustc.edu.cn; 2 Key Laboratory for Power Machinery and Engineering of MOE, Shanghai Jiao Tong University, Shanghai 200240, China; yell@sjtu.edu.cn

**Keywords:** C_3_H_2_F_3_Br, halon replacement, addition, elimination, Ab initio calculation, rate coefficients

## Abstract

CF_3_CBrCH_2_ (2-bromo-3,3,3-trifluoropropene, 2-BTP) is a potential replacement for CF_3_Br; however, it shows conflicted inhibition and enhancement behaviors under different combustion conditions. To better understand the combustion chemistry of 2-BTP, a theoretical study has been performed on its reactions with OH and H radicals. Potential energy surfaces were exhaustively explored by using B3LYP/aug-cc-pVTZ for geometry optimizations and CCSD(T)/aug-cc-pVTZ for high level single point energy refinements. Detailed kinetics of the major pathways were predicted by using RRKM/master-equation methodology. The present predictions imply that the –C(Br)=CH_2_ moiety of 2-BTP is most likely to be responsible for its fuel-like property. For 2-BTP + OH, the addition to the initial adduct (CF_3_CBrCH_2_OH) is the dominant channel at low temperatures, while the substitution reaction (CF_3_COHCH_2_ + Br) and H abstraction reaction (CF_3_CBrCH + H_2_O) dominates at high temperatures and elevated pressures. For 2-BTP + H, the addition to the initial adduct (CF_3_CBrCH_3_) also dominates the overall kinetics at low temperatures, while Br abstraction reaction (CF_3_CCH_2_ + HBr) and β-scission of the adduct forming CF_3_CHCH_2_ + Br dominates at high temperatures and elevated pressures. Compared to 2-BTP + OH, the 2-BTP + H reaction tends to have a larger effect on flame suppression, given the fact that it produces more inhibition species.

## 1. Introduction

The ozone depleting substances (ODSs) are man-made chemicals emitted into the atmosphere that deplete the stratospheric ozone layer. Up to the present day, many ODSs listed in the Montreal Protocol have been gradually phased out [1,2]. One of the major ODSs, CF_3_Br (bromotrifluoromethane, Halon 1301), which is a very effective fire suppressant, has been banned from production due to its high potential for ozone depletion, although it makes up to 80% of all the suppressants of cargo-bay fire suppression [3]. With the urgent need for fire suppression, a wide and international effort has been made to search for environmentally-safer halon replacements. The suitable candidates for halon should be satisfied with both a low global warming potential (GWP) and zero stratospheric ozone depletion potential (ODP) [1]. Unsaturated bromo-olefins are being considered as the potential replacements as they have an atmospheric lifetime of days to weeks and can be removed from the atmosphere by reacting with OH radical [1,4,5,6].

Among bromo-olefins, CF_3_CBrCH_2_ (2-bromo-3,3,3-trifluoropropane, 2-BTP) has received great attention because of its low ODP and GWP values of 0.0028–0.0052 and 0.23–0.26, respectively [4]. A great amount of studies have been focused on the inhibition chemistry mechanism, minimum extinguishing concentration, and combustion enhancement/suppression of 2-BTP by using theoretical calculation, model simulation or experiment [5,6,7,8,9,10,11,12,13,14,15,16,17]. According to previous studies, 2-BTP was considered to have a similar suppression performance to CF_3_Br given that both molecules contain a bromine atom and a CF_3_ group [7,11]. Unfortunately, 2-BTP failed in the mandated FAA aerosol can test (FAA-ACT), like other potential replacements, i.e., C_2_HF_5_ (HFC-125) and C_6_F_12_O (Novec 1230) [7,11,18]. In order to better understand why it promoted a higher overpressure when added at sub-inerting concentration and then reduced during the test, Babushok and Burgess et al. developed a chemical kinetic mechanism to describe the flame inhibition chemistry of 2-BTP [10,11]. However, in their model, the unimolecular decomposition and radical-induced decomposition (notably by OH and H) pathways for 2-BTP were incomplete and most of the kinetic data were obtained from estimation. Using the chemical model developed, Babushok and Linteris et al. [9,10,11] conducted numerical simulations on the premixed flames, thermodynamic equilibrium, and perfectly stirred-reactors to explore the inhibition and enhancement performance for 2-BTP and other potential replacements under wide combustion conditions. Their studies indicated that the enhancement phenomenon for 2-BTP, when added to lean hydrocarbon-air mixtures, results from the fuel-like property and the increase of the heat release, and the reactions of 2-BTP with OH and H radicals play a significant role in combustion chemistry [9,10,11]. For instance, almost 80% of the 2-BTP is consumed by reacting with H radical for a stoichiometric CH_4_-air flame with 1% loading, forming the inhibition species (CF_3_, Br and HBr) and hydrocarbons [11]. In addition, the equilibrium volume fraction of OH increases by a factor of 21, with 3% loading of 2-BTP for lean C_3_H_8_-air flames (Ф = 0.53), where 2-BTP shows the fuel effect [9]. Furthermore, Takahashi et al. [8] studied the effects of 2-BTP and others on the structure of diffusion flames and found that, while the agent weakened the premixed-like flame stabilizing base, the combustion enhancement occurred mainly in the trailing diffusion flame, primarily due to highly exothermic COF_2_ and HF formation reactions. Additionally, 2-BTP, as a potential fire suppressant, is directly emitted into the atmosphere to extinguish a fire, where an abundance of OH radical exists. This means that the reaction mechanism of 2-BTP with OH is significant for atmospheric chemistry [1]. However, the existing results could not explain which element or function group is responsible for the fuel-like property and which exothermic reaction enhances heat release and accelerates combustion. A systematical kinetic study is certainly needed to further improve the detailed mechanism.

The present study aims to fundamentally investigate the detailed kinetics for reactions of 2-BTP with OH and H radicals under combustion condition, and to provide evidence for the fuel-like property and the inhibition effect of 2-BTP. Reactions of 2-BTP with OH and H radicals include three mechanisms, which are abstraction, substitution, and addition followed by the subsequent decomposition. The reaction pathways were exhaustively explored by using high-level Ab initio quantum calculations. Kinetics for the major reactions on potential energy surfaces were calculated with the transition-state-theory-based master-equation technique. In particular, the variational transition state theory was used to compute the kinetic data for the barrierless reactions without well-defined saddle points.

## 2. Results and Discussion

**Figure 1:**
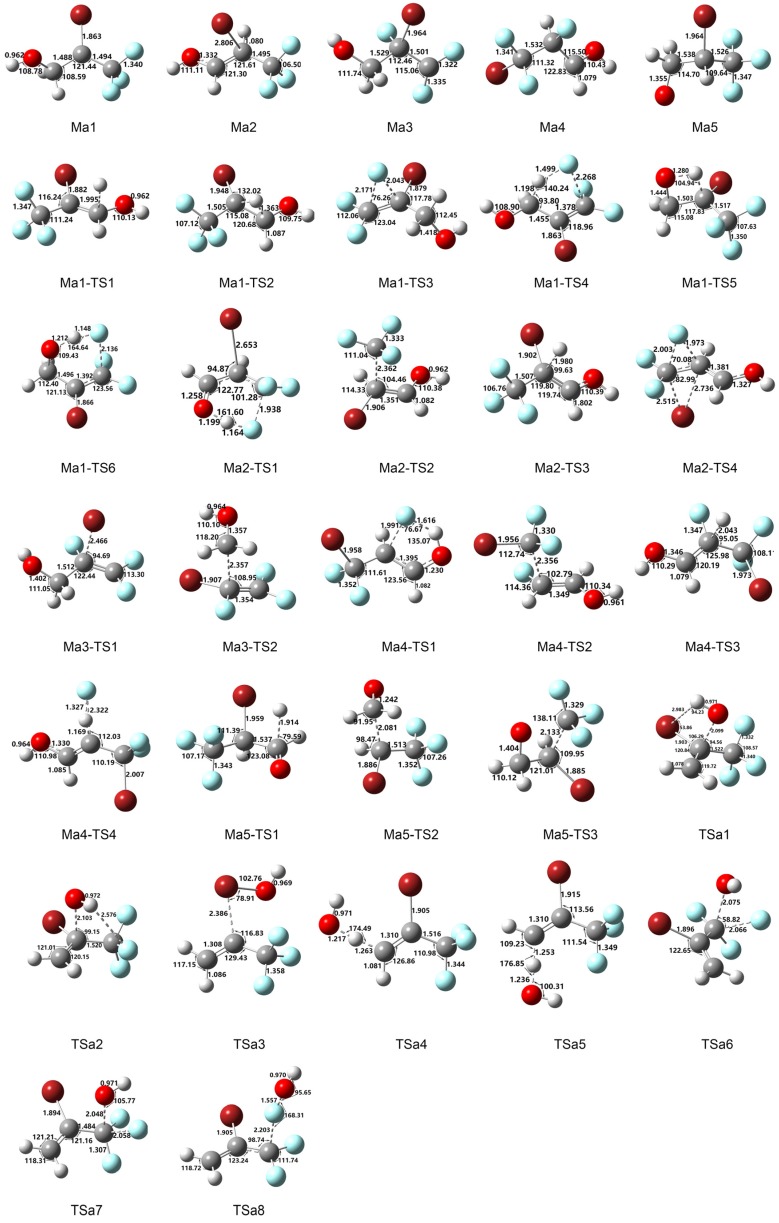
Optimized geometries of intermediates and transition states in 2-BTP + OH reaction calculated at B3LYP/aug-cc-pVTZ. Bond lengths and angles are in angstrom and degree, respectively.

**Figure 2:**
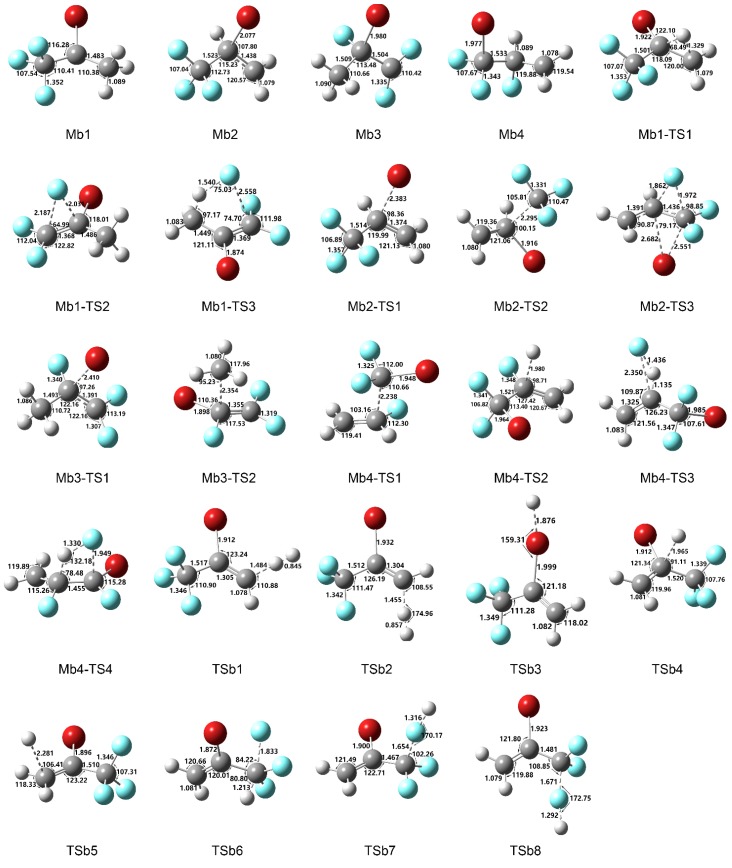
Optimized geometries of intermediates and transition states in 2-BTP + H reaction calculated at B3LYP/aug-cc-pVTZ. Bond lengths and angles are in angstrom and degree, respectively.

The intermediates, complexes, and products are labeled by the combination of letter (M, CR, and P) and number, with the letter a or b added in the middle to distinguish species involved in 2-BTP + OH or 2-BTP + H reactions. The optimized geometries of intermediates and transition states for these two reaction systems are depicted in Figure 1 and Figure 2, with geometries of other species illustrated in Figures S1 and S2 of Supplemental Material I. The zero-point corrected energies of all the stationary points at 0 K in kcal/mol are listed in Tables S1 and S2 of Supplemental Material I for 2-BTP + OH and 2-BTP + H, respectively. The Cartesian coordinates and harmonic frequencies are provided in Supplemental Material II.

### 2.1. Abstraction and Substitution

#### 2.1.1. CF_3_CBrCH_2_ + OH

**Figure 3:**
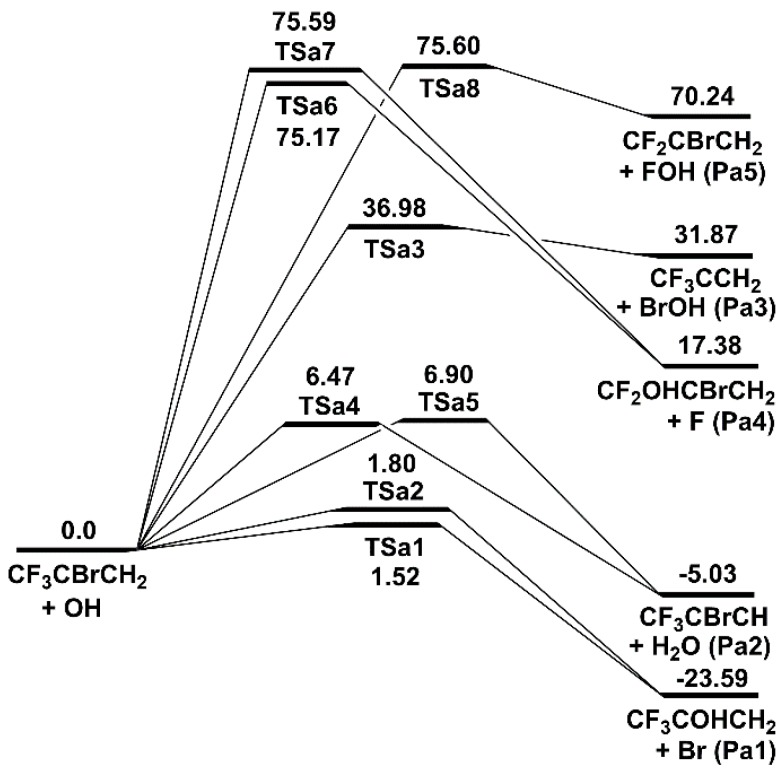
Schematic energy diagram of abstraction and substitution channels for the 2-BTP + OH reaction at 0 K in kcal/mol at CCSD(T)/aug-cc-pVTZ//B3LYP/aug-cc-pVTZ (zero-point vibrational energy included).

The abstraction and substitution channels of 2-BTP + OH are shown in Figure 3. For 2-BTP molecule, the three types of atoms (H, F, and Br) can be attacked by OH radical and undergo abstraction reactions. The H abstraction reactions proceed by initial formation of the van der Waals complexes (CRa4 and CRa5) and subsequent abstraction of H atom. For these specific channels, the complete energy diagram containing the van der Waals complexes is shown in Figure S3 of Supplemental Material I. The energies of the two transition states, TSa4 and TSa5, are 6.47 and 6.90 kcal/mol, respectively, computed at CCSD(T)/aug-cc-pVTZ//B3LYP/aug-cc-pVTZ. These energies are very similar to the values of 6.84 and 7.58 kcal/mol at CCSD(T)/aug-cc-pVDZ//B3LYP/6-311++G(d, p) by Chen et al. [4]. 1-BTP, as an isomer for 2-BTP, also has two H abstraction channels with 11.1 and 13.9 kcal/mol barrier heights, respectively, at CCSD(T)/aug-cc-pVDZ//B3LYP/6-311++G(d, p) by Zhang et al. [5]. It can be seen that the presence of Br atom in central carbon atom more facilitates H abstractions. The Br abstraction at the central carbon occurs via the transition state of TSa3, which is about 30 kcal/mol higher than the transition states of H-abstractions. This Br abstraction was not considered in the study by Chen et al. [4], while Zhang et al. [5] reported Br abstraction for 1-BTP with a high barrier 42.3 kca/mol. This is of great interest when one realizes that the C-Br bond is the weakest bond in 2-BTP [6,10]. It can be explained by the fact that the interaction between the nucleophilic OH and the electrophilic H is much stronger than that between OH and Br (halogens are normally nucleophilic). The F abstraction proceeds via a rather high energy barrier, i.e., TSa8 with 75.60 kcal/mol, which is in accordance with the previous result with 75.50 kcal/mol [4]. This results from the combined effect of the weak interaction between F and OH and the strong bonding of C–F. Among the three types of abstractions, the H abstractions are the only exothermic reactions and happen with the lowest energy barriers.

Two classes of substitution reactions are identified for the reactions of 2-BTP with OH. In the first class, OH radical attacks the carbon atom in CF_3_, leading to substitution reactions though the transition states of TSa6 and TSa7. Note that the presence of two transition states results from the different attacking directions of OH radical. In the other class, OH radical attacks the central carbon atom, leading to direct substitution reactions though TSa1 and TSa2 instead of forming an initial adduct. The absence of the adduct here is because the addition of OH radical, which is highly electronegative, further weakens the C-Br bonding strength, so that Br and OH cannot co-exist on the central carbon. Both TSa1 and TSa2 contain hydrogen bonding in their molecular structures, formed from the interaction between the H atom in OH moiety and Br (for TSa1) or F (for TSa2). For Br substitution, Chen et al. [4] only reported one transition state with a higher barrier (i.e., 3.18 kcal/mol), and Zhang et al. [5] reported two transition states with 1.5 and 2.1 kcal/mol for 1-BTP, of which the energies were obtained at CCSD(T)/aug-cc-pVDZ//B3LYP/6-311++G(d, p). According to our calculations accompanied with the results of previous works [4,5], Br substitutions have the lowest energy barriers among the bimolecular reactions directly producing two molecular products. Additionally, for all the substitutions, the two channels via TSa1 and TSa2 are the exothermic reactions.

One might think about the substitution reaction that proceeds by OH attacking the carbon atom in CH_2_. As is shown later in Section 2.2.1., when OH attacks this specific carbon atom, it is the addition mechanism instead of substitution that takes place.

#### 2.1.2. CF_3_CBrCH_2_ + H

**Figure 4:**
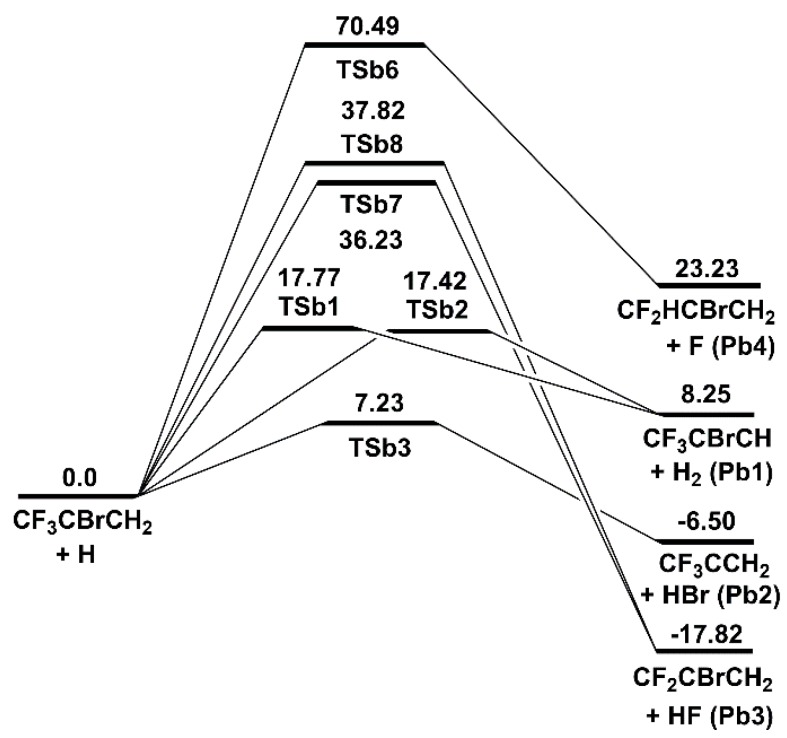
Schematic energy diagram of abstraction and substitution channels for the 2-BTP + H reaction at 0 K in kcal/mol at CCSD(T)/aug-cc-pVTZ//B3LYP/aug-cc-pVTZ (zero-point vibrational energy included).

A total of five abstraction channels are identified for the 2-BTP + H reaction, as shown in Figure 4. Most of the reactions proceed via initially forming the van der Waals complexes (i.e., CRb1, CRb2, CRb3, and CRb8). These prereaction complexes actually have no impact on the final kinetic results. Nevertheless, the complete energy diagram with the van der Waals complexes is provided in Figure S4 of Supplemental Material I. The H abstractions proceed by the transition states of TSb1 and TSb2, which are 17.77 and 17.42 kcal/mol above the reactants, respectively. These energy barriers are higher by over 10 kcal/mol than the H abstractions in 2-BTP + OH. The Br abstraction proceeds via an energy barrier of 7.23 kcal/mol (TSb3), which is about 30 kcal/mol lower than the barrier of the counterpart reaction in 2-BTP + OH. This can be easily attributed to the strong interaction between the electrophilic H and the nucleophilic Br. The F abstractions occur via the transition states of TSb7 and TSb8, with the energies being 36.23 and 37.82 kcal/mol above the reactants, respectively. These two channels make little contribution to the whole mechanism due to the high barriers.

With respect to the substitution, there is only one channel being identified, which results from H attack to the carbon atom in CF_3_ group and produces CF_2_HCBrCH_2_ + F. This channel proceeds via the transition state of TSb6, with the energy of 70.49 kcal/mol. Due to the high barrier, this channel can be safely neglected. H attack to the other two carbons basically leads to the additions, where the initial adducts are initially formed followed by decompositions, as will be seen later in Section 2.2.2.

### 2.2. Addition Mechanism

#### 2.2.1. CF_3_CBrCH_2_ + OH

**Figure 5:**
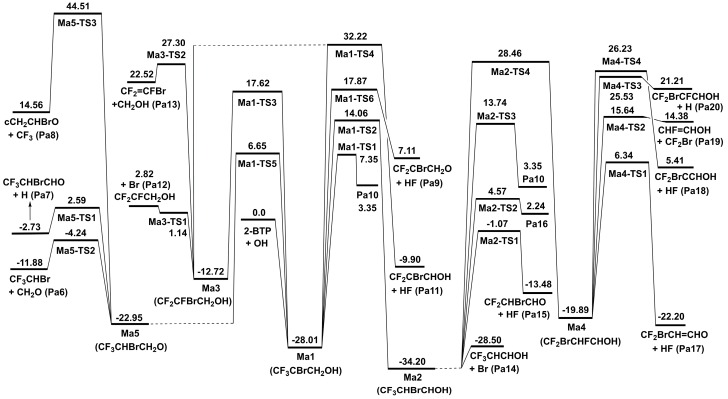
Schematic energy diagram of addition and eliminations for 2-BTP + OH at 0 K in kcal/mol at CCSD(T)/aug-cc-pVTZ//B3LYP/aug-cc-pVTZ (zero-point vibrational energy included).

The potential energy surface for addition and subsequent decomposition channels is provided in Figure 5 for 2-BTP + OH. The OH addition reaction happens at the carbon site in CH_2_ and forms the initial adduct, Ma1 (CF_3_CBrCH_2_OH). Note that OH addition to the remaining carbons simply leads to the substitution reactions, as discussed in Section 2.1.1. The addition process of 2-BTP + OH = Ma1 is a barrierless reaction without a well-defined saddle point. The study by Chen et al. [4] has located three stable intermediates formed from different orientations of OH radical. In the present work, only the most stable one (i.e., Ma1) is considered, and the partition functions of the other isomers are taken into account by including the internal rotation treatment of OH group in Ma1.

Subsequent reactions of Ma1 mainly include dissociation and isomerization. Two of the dissociation channels proceed via β-scissions, i.e., the β-scission of C–H bond with the lowest barrier of Ma1-TS1 and the β-scission of C–F with Ma1-TS4. The C–F β-scission differs from the C–H one in that the highly nucleophilic F atom directly takes the H atom away during its departure from the carbon atom, forming the products of CF_2_CBrCHOH + HF. There is a third dissociation channel that occurs through the transition state of Ma1-TS6 leading to CF_2_CBrCH_2_O + HF. The isomerization of Ma1 gives rise to three different isomers. The one forming Ma5 via the transfer of H atom in OH to the central carbon has the lowest isomerization barrier, Ma1-TS5. This four-membered ring transition state lies 6.65 kcal/mol above the initial reactants. The Ma2 and Ma3 isomers are formed via relatively high barriers, Ma1-TS2 and Ma1-TS3, respectively. These three-membered ring transition states represent the transfer of H or F atom at adjacent carbons to the central carbon.

The β-scissions are the most important reactions for the subsequent decomposition of Ma2, Ma3 and Ma5 isomers. For Ma2 decomposition, the β-scission of the C–Br bond, which is a barrierless reaction here, is the most favored channel and forms the products of CF_3_CHCHOH + Br. The β-scissions of C–C and C–H bonds are much less competitive, occurring with the transition states of Ma2-TS2 (4.57 kcal/mol) and Ma2-TS3 (13.74 kcal/mol), respectively. The O–H bond can also undergo the β-scission reaction, but the leaving H atom readily abstracts the F atom in the CF_3_ group to form CF_2_CHBrCHO + HF. This channel proceeds via a relatively low barrier, Ma2-TS1, with the energy of −1.07 kcal/mol. Apart from the β-scissions, there is an isomerization reaction that is of special interest. This special reaction proceeds by the interchange of F and Br atoms at two adjacent carbons in a single step, leading to the formation of Ma4 isomer via Ma2-TS4. Although this type of isomerization plays an important role in the saturated halogenated systems [19,20,21], it is unlikely to happen here for Ma2 considering the high barrier of 62.66 kcal/mol.

As for Ma4, four decomposition channels were identified. Two of them are the β-C–C and β-C–H scissions though the transition states of Ma4-TS2 and Ma4-TS3, respectively. The other two are the HF elimination reactions via the transition states of Ma4-TS4 and Ma4-TS1, respectively. For Ma3, there are three decomposition channels, including the β-scissions of C–Br, C–C, and C–F bonds. Among them, the β-scission of the C–Br bond proceeding via Ma3-TS1 is the most favored channel. For Ma5 decomposition, the two β-scissions of C–C and C–H bonds via Ma5-TS2 and Ma5-TS1, respectively, are the most significant channels. The other channel forming the cyclic OCH_2_CHBr plus CF_3_ occurs via a very high barrier, Ma5-TS3, with the energy of 44.51 kcal/mol. This channel can be entirely ignored.

#### 2.2.2. CF_3_CBrCH_2_ + H

**Figure 6:**
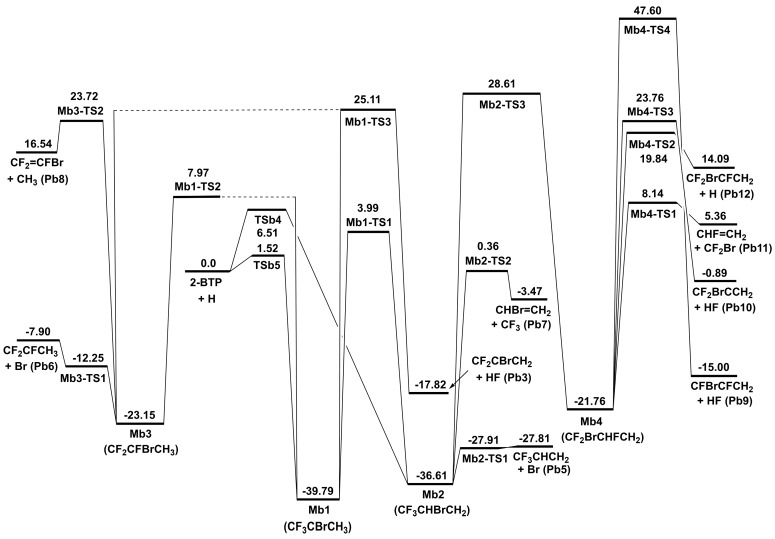
Schematic energy diagram of addition and eliminations for 2-BTP + H at 0 K in kcal/mol at CCSD(T)/aug-cc-pVTZ//B3LYP/aug-cc-pVTZ (zero-point vibrational energy included).

The potential energy surface for addition and subsequent decomposition channels is provided in Figure 6 for 2-BTP + H. The addition of H atom to the C=C double bond generates two stable intermediates, Mb1 and Mb2, via the transition states of TSb5 and TSb4, respectively. Recall that H addition to the carbon atom in CF_3_ leads to the substitution reaction, as discussed in Section 2.1.2. Note that TSb4 lies about 5 kcal/mol above TSb5, which results from the steric hindrance of CF_3_ moiety and Br atom connected to the central carbon [6]. The formation of Mb1 and Mb2 have the lowest barriers among all reactions from the bimolecular reactants. Subsequent reactions of Mb1 mainly include dissociation and isomerization. The dissociation reaction proceeds via the energy barrier of Mb1-TS3, producing CF_2_CBrCH_2_ + HF. This channel is expected to be unimportant due to the high barrier. One of the isomerization channels leads to the other initial adduct, Mb2, via the transition state of Mb1-TS1 (3.99 kcal/mol). The other isomerization channel forms Mb3 via a higher barrier of Mb1-TS2, which has the energy of 7.97 kcal/mol.

The β-scissions are the most significant reactions for further decomposition of Mb2 and Mb3. As for Mb2, its decomposition is dominated by the β-scission of the C–Br bond, which occurs via the energy barrier of Mb2-TS1. The β-scission of the C–C bond occurs via a much higher barrier of Mb2-TS2, lying over 28 kcal/mol above Mb2-TS1 for C–Br β-scission. The large difference in the energy barriers of these two channels can be easily attributed to the different chemical bond energies of C–C and C–Br. Similar to Ma2 in 2-BTP + OH, Mb2 can also undergo the interchanging of F and Br atoms via Mb2-TS3 to rearrange to Mb4. This channel has a rather high energy barrier, and is thus expected to have a negligible contribution to the overall kinetics. Following decomposition of Mb4 also mainly proceeds by the β-scission reactions. For Mb3 decomposition, β-scission of C-Br via Mb3-TS1 is the most dominant channel, followed by β-scissions of C–C and C–F bonds. Only the C–Br β-scission will be considered in the following kinetic calculations.

The discussions above suggest that the reactions to Mb1 and Mb2 followed by β-scission of the C–Br bond are expected to dominate the 2-BTP + H addition process.

### 2.3. Kinetic Calculations

**Scheme 1:**
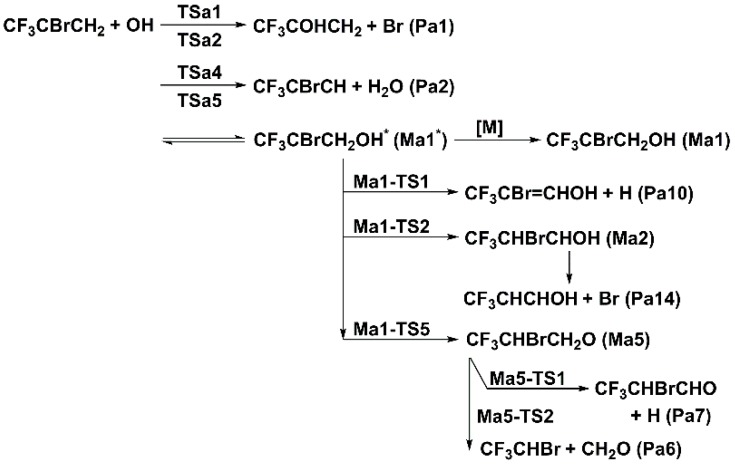
Major reaction channels involved in kinetic calculations of 2-BTP + OH. * indicates the energetically activated species.

**Scheme 2:**
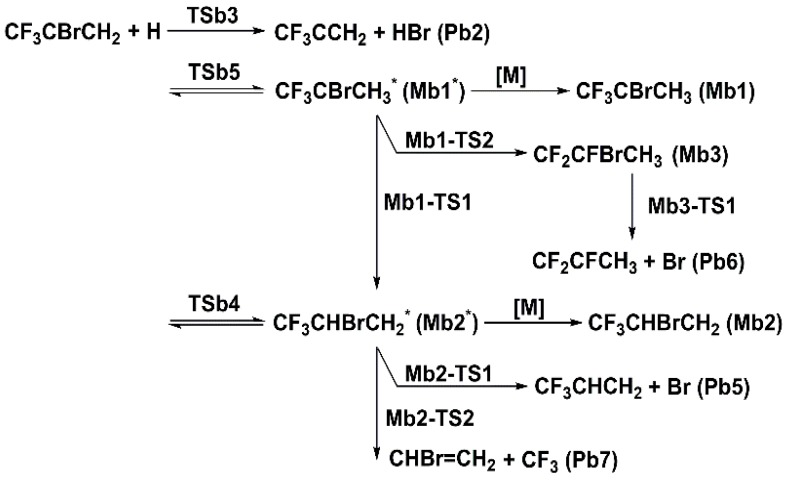
Major reaction channels involved in kinetic calculations of 2-BTP + H. * indicates the energetically activated species.

Based on the quantum chemical calculations, only the channels listed in Scheme 1 and Scheme 2 were considered in the kinetic calculations of 2-BTP + OH and 2-BTP + H reactions, respectively. According to our computing kinetic results, the ratios of computed rate constants are small (<2) when the value of ΔE_0_ varies within 150–200, and the value of n varies within 0.75–0.85 in the <ΔE_down_> = ΔE_0_(T/300)*^n^* form. Moreover, transition states Ma1-TS2 and Ma1-TS5 for 2-BTP + OH, and Mb1-TS1 for 2-BTP + H involved in H-migrations are affected by tunneling correction, and thus the rate coefficients of H-migrations and their competitive reactions for two computing systems are influenced below 1000 K.

#### 2.3.1. CF_3_CBrCH_2_ + OH

**Figure 7:**
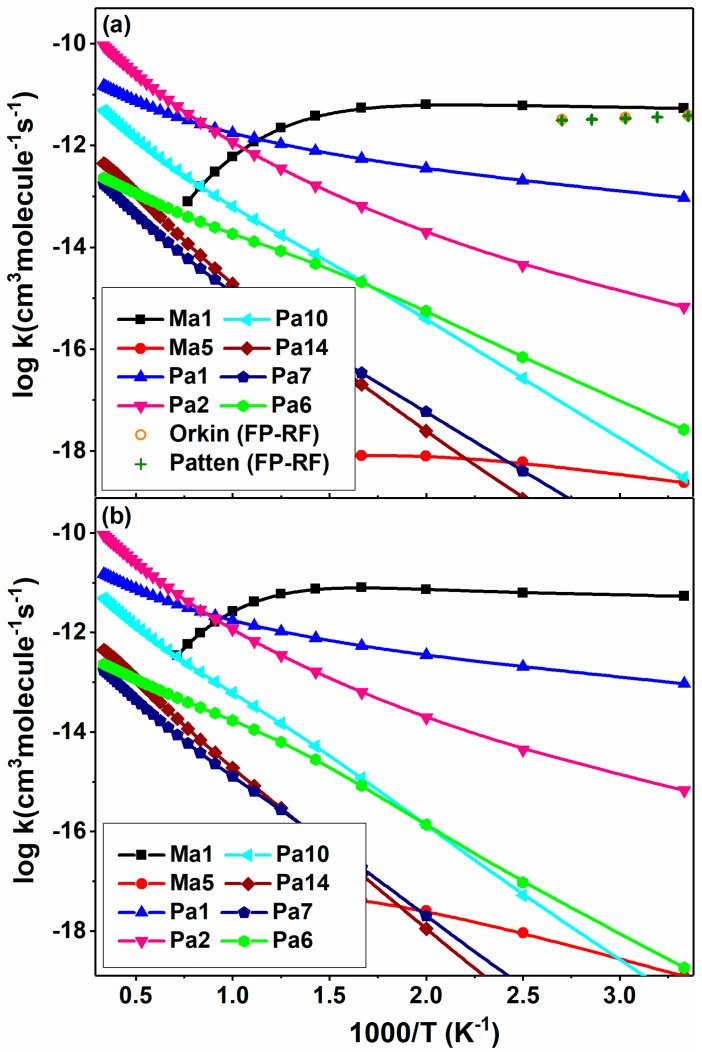
Rate constants of the major channels for 2-BTP + OH at 300–3000 K at the pressures of (**a**) 0.1 atm and (**b**) 1 atm in N_2_. Note: orange circles and olive crosses indicate the rate constants of 2-BTP + OH at 100 and 30 torr measured by Orkin et al. and Patten et al. respectively.

Figure 7 plots the temperature-dependent rate coefficients of the major channels, accompanied with the experimental data [6,22] at 300–3000 K at 0.1 and 1 atm in N_2_, with those at 100 atm provided in Figure S5 of Supplemental Material I. Our computed rate coefficients are slightly larger than these experimental data measured by using flash photolysis resonance fluorescence technique reported by Orkin et al. [6] and Patten et al. [22], but still are in consistent with them. As shown in Figure 7, the addition to Ma1 dominates the 2-BTP + OH reaction at low temperatures, while the H abstraction channel (Pa2) dominates at high temperatures. The substitution forming CF_3_COHCH_2_ + Br (Pa1) is also of great importance at medium to high temperatures. Orkin et al. proposed that the initial addition reactions of OH to the double bond in Br-containing fluorinated alkenes are two main degradation pathways in the atmosphere [6]. As seen in present work, Br substitution and the addition forming Ma1 have the largest rate coefficients comparing with the other pathways below 1000 K. The addition reaction exhibits a negative-temperature coefficient behavior, and the rate of decrease becomes slower at the higher pressure. This is because the unreactive collisions, which stabilize the energetically activated species, are more encountered at higher pressures and increase the stabilization of Ma1. Unlike the addition, the pressure effect on the rate constants of abstraction and substitution is very minor. The competitive behaviors of these two channels are controlled by the combined effect of enthalpy and entropy. At low temperature, the enthalpic effect dominates and the substitution channel is more favored, whereas at high temperature, the H abstraction channel is more favored because of the entropic effect.

From Figure 7a, the rate constants of the further dissociations of Ma5 are much faster than its other reactions above 400 K, which implies that the formed activated species of Ma5 will mostly decompose into separate products instead of being stabilized or reaction back to the reactants. At pressures high enough, collisions with bath gas get much more frequent, and there will be more stabilizations into the Ma5 well, such as the situation in Figure S5 of the Supplemental Material I.

**Figure 8:**
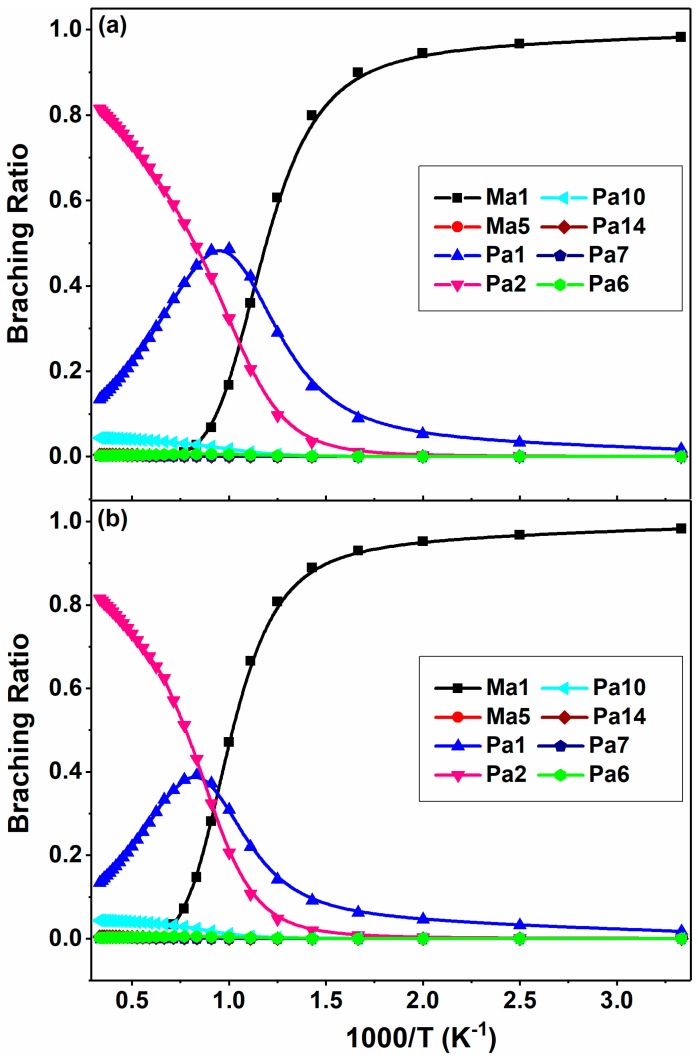
Branching ratios of the major channels for 2-BTP + OH at 300–3000 K at the pressures of (**a**) 0.1 atm and (**b**) 1 atm in N_2_.

The branching ratios for the major channels are shown in Figure 8 at 0.1 and 1 atm in N_2_. From this plot, the addition to Ma1 contributes almost exclusively to the 2-BTP + OH reaction at 300 K. As the temperature increases, the contribution of the addition reaction starts to decrease while that of H abstraction and substitution starts to increase dramatically. The substitution reaction, Pa1, makes the largest contribution at medium temperatures, e.g., 900–1150 K at 0.1 atm. After that, its yield starts to decrease and the H abstraction reaction, Pa2, becomes the most dominant channel. The β-scission channel, Pa10, has only a minor contribution to the overall kinetics, with the branching less than 5%. The situation at 1 atm is similar, except that the dominance of Ma1 is extended to higher temperature and the relative yield of substitution reaction becomes smaller.

**Figure 9:**
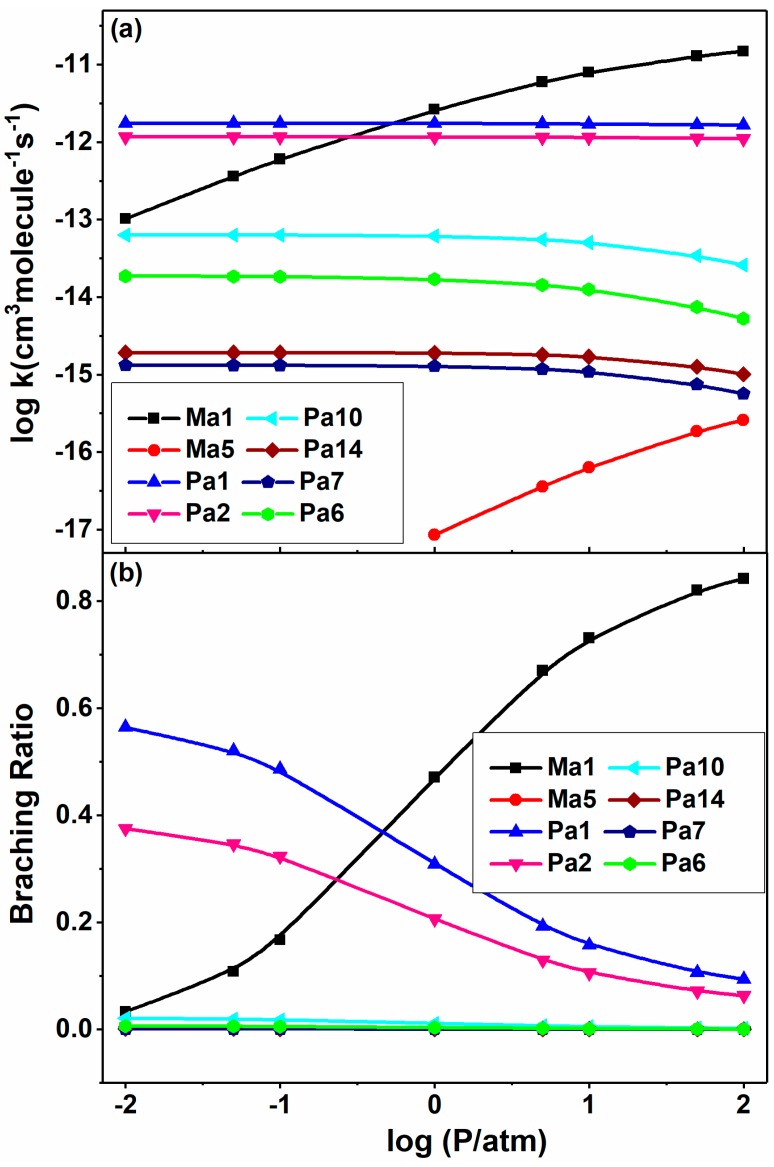
Pressure-dependent rate constants (**a**) and branching ratios (**b**) of the major channels for 2-BTP + OH at 0.01–100 atm in N_2_ at 1000 K.

Furthermore, the rate coefficients and branching ratios as a function of pressure are demonstrated in Figure 9 for the major channels at 1000 K. From Figure 9a, it can be seen clearly that the H abstraction (Pa2) and substitution (Pa1) reactions vary little with pressure. At this temperature, these two channels dominate the 2-BTP + OH reaction below 1 atm, and the substitution channel is more favored than the abstraction over the entire pressure range. The addition channel (Ma1) dominates the high-pressure kinetics, and its rate constants increase with the pressure. It is worth noting that the rate constants of the β-scission channels, Pa6, Pa7, Pa10 and Pa14, are also insensitive to the pressure under investigated conditions. These product channels can be considered as formally direct pathways, where the reactants traverse a sequence of transition states without being stabilized into the intermediate wells.

Among the four major channels of 2-BTP + OH reaction, the channel of Pa10 is endothermic, but it has only a minor contribution to the overall kinetics. The other three pathways (particularly Ma1 and Pa1) are exothermic and have a large exothermic effect at low to medium temperatures where Ma1 and Pa1 dominate. Although Pa1 generates a Br atom, which has the flame inhibition effect, it is not the most favored channel for 2-BTP + OH reaction at 1 atm. Moreover, the three most important channels, i.e., the addition of Ma1, the substitution of Pa1, and the abstraction of Pa2, all arise from the reactions of –C(Br)=CH_2_ moiety with OH radicals. This drives us to think that it is the –C(Br)=CH_2_ moiety that is likely to be responsible for the fuel-like property of 2-BTP when reacting with OH radicals. Additionally, in the inhibition chemistry model of 2-BTP [10], only H abstraction and substitution channels were considered for the 2-BTP + OH reaction, with their rate constants simply from estimations. The kinetic results in the present work should be used for the update and improvement of the existing model.

#### 2.3.2. CF_3_CBrCH_2_ + H

**Figure 10:**
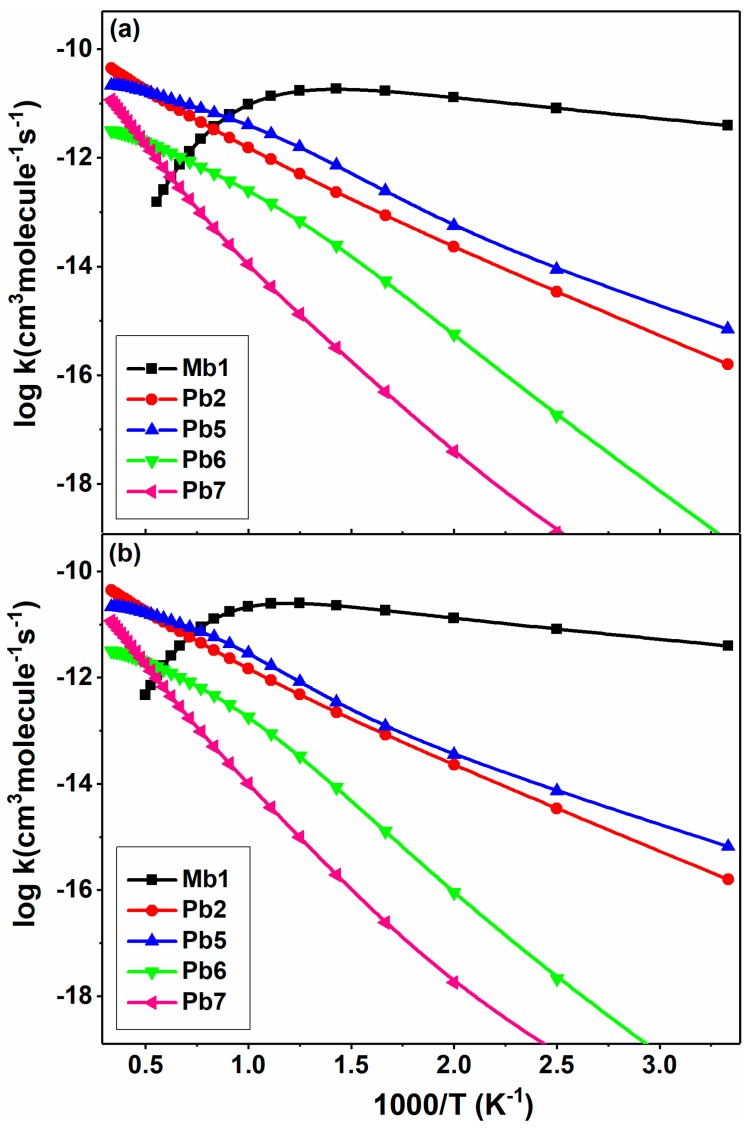
Temperature-dependent rate constants of major channels for 2-BTP + H at 300–3000 K at the pressures of (**a**) 0.1 atm and (**b**) 1 atm in N_2_.

Figure 10 plots the temperature-dependent rate constants of five major channels for the reactions of 2-BTP with H at 0.1 and 1 atm in N_2_. It can be seen that the addition to Mb1 dominates the overall reaction below 1100 K at 0.1 atm and 1300 K at 1 atm, while the Br abstraction (Pb2) and the β-scission (Pb5) are more competitive at high temperatures. Compared to Pb5, the other β-scissions, Pb6 and Pb7, are orders of magnitude slower over 300–3000 K. At 0.1 atm, the Mb1 channel shows a negative-temperature coefficient behavior, of which the rate constants first increase below 900 K and then decrease with temperature. This increase behavior becomes more obvious at even higher pressures, as shown in Figure S6 of the Supplemental Material I. This is because the Mb1 well is rather deep, and so, at higher pressures, there tends to be more stabilization into the well and less reaction to other products compared to the situation at lower pressures. As the most important channel for the subsequent consumption of Mb1, the decline of rate constants of Pb5 at higher pressures further validates the explanation above.

**Figure 11:**
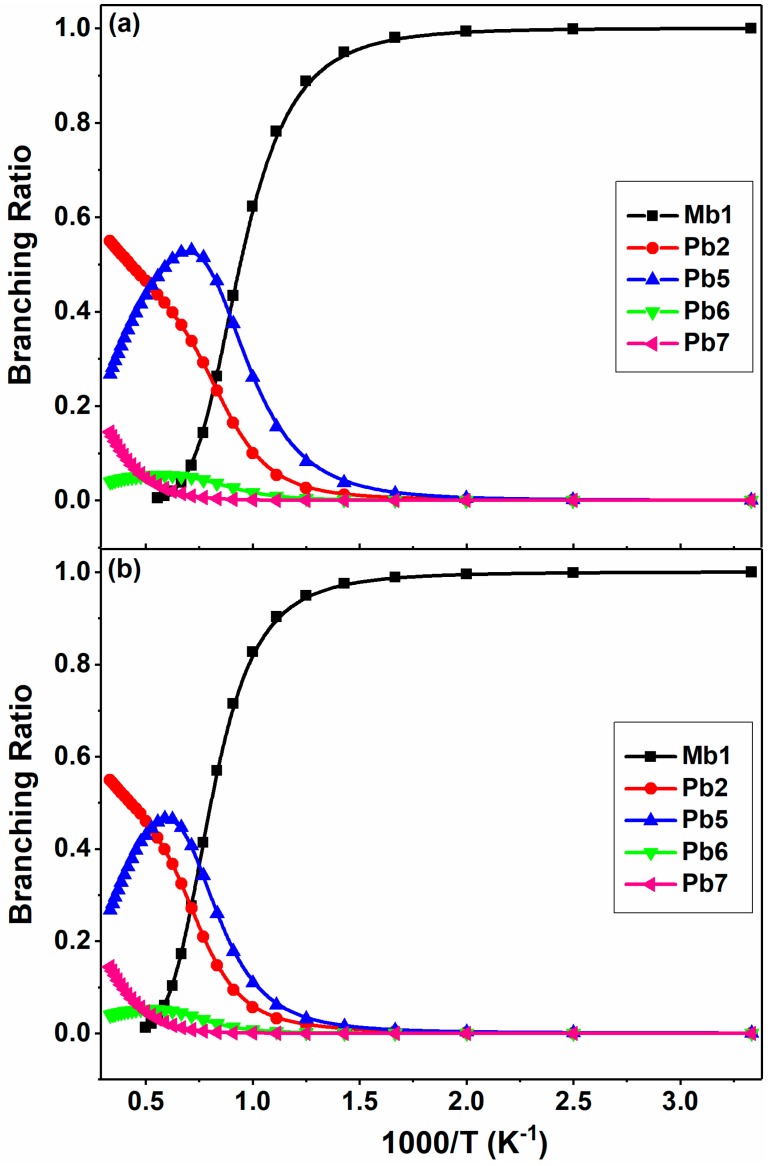
Branching ratios of major channels for 2-BTP + H at 300–3000 K at the pressures of (**a**) 1 atm and (**b**) 100 atm in N_2_.

The branching ratios of the major channels are shown in Figure 11 at 0.1 and 1 atm. At 0.1 atm, the addition to Mb1 overwhelmingly dominates the overall kinetics below 1100 K. As the temperature goes up, the yield of the β-scission channel (Pb5) increases rapidly, and becomes the most important channel between 1100–1900 K with a branching of 0.37–0.53. Note that its yield reaches a maximum value at around 1400 K, and then decreases after that. The Br abstraction channel (Pb2) has the largest contribution at higher temperatures. The other two β-scissions (Pb6 and Pb7) make only a minor contribution to the overall reaction, but still cannot be neglected especially at high temperatures. The situation is similar at 1 atm. The major difference lies in that the dominance of Mb1 is extended to 1400 K and the relative yield of Pb5 becomes smaller.

**Figure 12:**
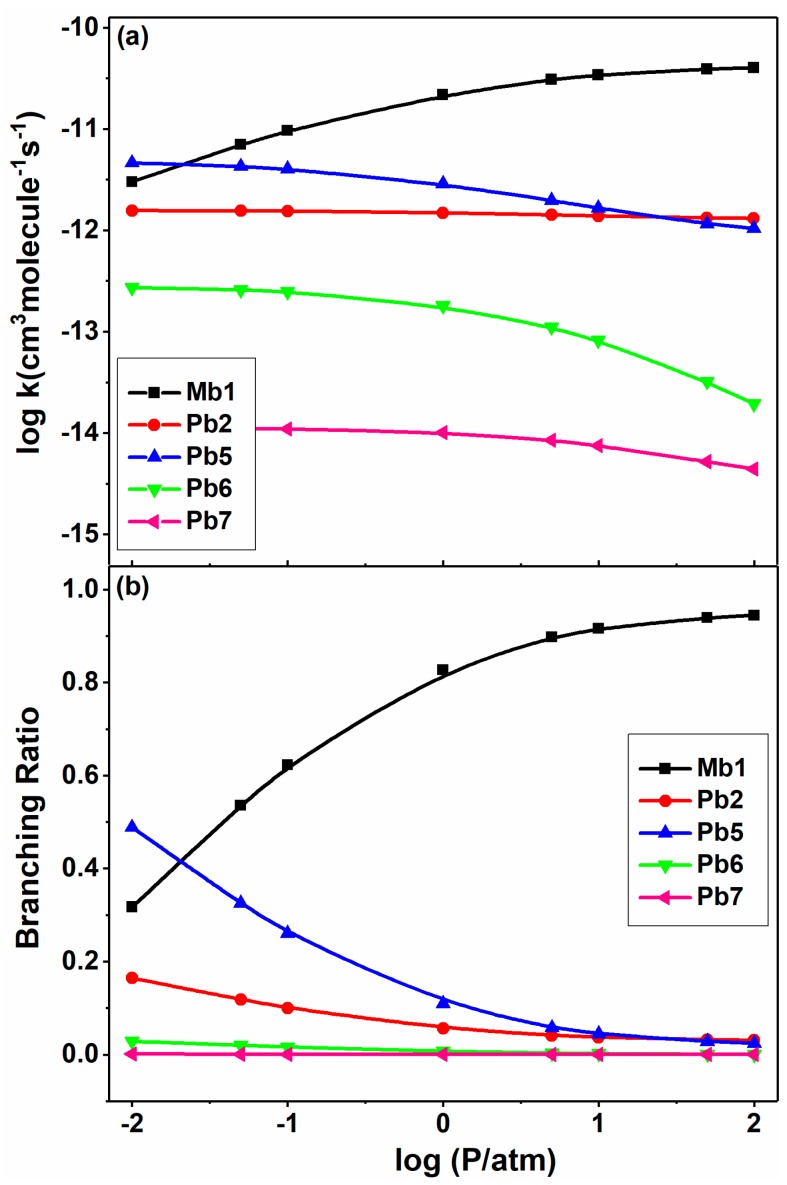
Pressure-dependent rate constants (**a**) and branching ratios (**b**) of major product channels for 2-BTP + H at 0.01–100 atm at 1000 K in N_2_.

In addition, the rate coefficients and branching ratios are demonstrated in Figure 12 as a function of pressure for the five main channels in 2-BTP + H reaction at 1000 K. As expected, the rate constants of Mb1 and Pb5 channels exhibit an opposite behavior in variation with the pressure, as shown in Figure 12a. The Mb1 channel makes the largest contribution for most of the pressures studied, with a yield of 0.94 at 100 atm at this temperature. The Br abstraction (Pb2) are insensitive to the pressure, and the rate constants remain almost unchanged at different pressures. The rate constants of Pb6 and Pb7 also demonstrate an inverse variation of Mb1, especially at higher pressures, again verifying the role of pressure in systems with wells.

According to the quantum chemical calculations, the five major channels for 2-BTP + H reaction are all exothermic. Among them, the addition channel and the β-scission channel are the most exothermic, with Mb1 and Pb5 lying 39.79 and 27.81 kcal/mol, respectively, below the reactants. However, four of the five major channels generate a large amount of Br, HBr, and CF_3_ species, and these species play a key role in flame inhibition. The previous model [10] has included these channels except the one of Pb6 forming Br plus CF_2_CFCH_3_. The present results offer useful data for the improvement of the previous model. Moreover, the major pathways all result from the reactions between the –CBr=CH_2_ moiety and H radical. This implies that, it is the –CBr=CH_2_ moiety that is mostly likely to be responsible for the fuel-like property of 2-BTP when reacting with H radicals.

## 3. Theoretical Methodology

Suggested by Nizovtsev et al., the B3LYP/aug-cc-pVTZ method can predict the geometry parameters, vibrational frequencies and rotational constants with good accuracy for fluorine- and bromine-containing systems [23]. Therefore, in this work, the equilibrium geometries and vibrational frequencies of all stationary points were computed at the B3LYP/aug-cc-pVTZ level [24,25,26,27,28]. Intrinsic reaction coordinate (IRC) calculations were conducted at the same level to ensure that the transition states (TSs) connect the desired reactants and products. The analysis of vibrational frequencies confirmed that all transition states are characterized by the single imaginary frequency, and all reactants, products, and complexes have only positive frequencies. Afterwards, the CCSD(T) method, i.e., coupled-cluster method with single and double excitations and perturbative triples corrections [29,30], was applied for the high-level single-point energies with the aug-cc-pVTZ basis set. For barrierless channel without a well-defined saddle point, the B3LYP/aug-cc-pVTZ method was used to compute the minimum energy potential (MEP) between two interacting fragments with a grid 0.05 Å of 1.4–5.4 Å. For the points within the separation of 1.8–2.5 Å, CCSD(T)/aug-cc-pVTZ method was performed to obtain more accurate interaction energies. Additionally, the spin-orbit correction (3.51 kcal/mol) were considered into the C–Br bond dissociations involved in 2-BTP reacting with OH and H, which were estimated by taking the experimental spin-orbit splitting for Br (^2^P_3/2_ and ^2^P_1/2_) [23,31,32,33]. Quantum chemical computations were carried out with the Gaussian 09 package [34].

Based upon PESs for 2-BTP with OH and H radicals, temperature- and pressure-dependent phenomenological rate coefficients for several major channels were computed by using RRKM/master-equation methodology. For the channels without well-defined saddle points, the energy-resolved variational transition state theory (µVTST) was used. In addition, the internal torsional modes were assumed as one-dimensional hindered rotors, the hindrance potentials of which were obtained by the relaxed scan with an increment of 10 degree at B3LYP/aug-cc-pVTZ. The asymmetric Eckart model was used to correct the tunneling effect [35,36]. The collisional energy transfer was described by a single exponential-down model. For the average energy transferred per collision, <ΔE_down_>, there is no experimental date available for the target systems in the present work. Vahid et al. [37] used a value of 129 cm^−1^ for CF_3_CF=CF_2_ reacting with OH, and Zhang et al. [38] used a value of 200 cm^−1^ in N_2_ and 100 cm^−1^ in Ar for the reactions of CF_3_CH=CH_2_ with OH. Considering a heavy Br atom in 2-BTP, we used a temperature-dependent form for this value, that is, <ΔE_down_> = ΔE_0_(T/300)*^n^* (ΔE_0_ = 200, *n* = 0.85). The interaction between reactants and bath gas was modeled by the Lennard-Jones (L-J) potential. The L-J parameters were estimated by using the Benson method: σ = 5.20 Å, ε = 528 K for 2-BTP with OH, and σ = 5.08 Å, ε = 448 K for 2-BTP with H [39,40]. In this present work, we used three buffer gases (Ar, Kr, and N_2_) to calculate rate constants, which facilitates the comparison and application of the kinetic data. The L-J parameters for these three buffer gases are recommended by Hippler et al. [41]: σ = 3.47 Å, ε = 114 K for Ar, σ = 3.66 Å, ε = 178 K for Kr and σ = 3.74 Å, ε = 82 K for N_2_. The kinetic predictions were performed with the MESS program, which well deals with the complex reaction mechanisms including multiple wells and multiple channels [22,42,43,44].

## 4. Conclusions

In the present work, an exhaustive exploration for the potential energy surfaces has been performed for the reactions of 2-BTP with OH and H radicals at CCSD(T)/aug-cc-pVTZ//B3LYP/aug-cc-pVTZ. After that, detailed kinetics of the major pathways have been computed with the RRKM/master-equation method. Quantum chemical calculations and kinetic predictions show that, at low temperatures, the addition to the initial adducts is the most dominant channel for both reaction systems, and the dominance is extended to higher temperatures with increasing pressure. At high temperatures and elevated pressures, the H abstraction and the Br substitution dominate the kinetics of the 2-BTP + OH reaction, while the Br abstraction and the β-scission of adduct isomer, which yields CF_3_CHCH_2_ and Br, dominate the kinetics of the 2-BTP + H reaction. The channels of the most significance all result from the reactions between the –C(Br)=CH_2_ moiety and OH or H radicals, indicating that the –C(Br)=CH_2_ moiety is most likely to be responsible for the fuel-like property of the 2-BTP molecule. For the 2-BTP + OH reaction, only one major channel generates the inhibition species (Br), whereas, for the 2-BTP + H reaction, there are four important pathways to generate the inhibition species (Br, HBr, and CF_3_). This drives us to conclude that the reaction of 2-BTP with H radicals has a larger contribution to the flame inhibition than its reaction with OH radicals.

## Supplementary Materials

The following are available online:

**Table:**

Order	Filename	Content
**1**	**Supplemental Material-I**	(1) Geometries of reactants, products and prereaction complexes involved in the reactions of 2-BTP + OH. (2) Geometries of reactants, products and prereaction complexes involved in the reactions of 2-BTP + H. (3) Complete potential energy diagram for channels including prereaction complexes in the reactions of 2-BTP + OH. (4) Complete potential energy diagram for channels including prereaction complexes in the reactions of 2-BTP + H. (5) Temperature-dependent rate constants (a) and branching ratios (b) of the major channels for 2-BTP + OH at 100 atm in N_2_. (6) Temperature-dependent rate constants (a) and branching ratios (b) of the major channels for 2-BTP +H at 100 atm in N_2_. (7) Relative energies of all the stationary points involved in 2-BTP + OH reaction at 0 K in kcal/mol by using the CCSD(T)/aug-cc-pVTZ//B3LYP/aug-cc-pVTZ method. (8) Relative energies of all the stationary points involved in 2-BTP + H at 0 K in kcal/mol by using the CCSD(T)/aug-cc-pVTZ//B3LYP/aug-cc-pVTZ method.
**2**	**Supplemental Material-II**	Cartesian coordinates and frequencies of all the species involved in the present work.
